# Geospatial analysis for reproductive, maternal, newborn, child and adolescent health: gaps and opportunities

**DOI:** 10.1136/bmjgh-2019-001702

**Published:** 2019-07-01

**Authors:** Zoe Matthews, Barbara Rawlins, Jennifer Duong, Yordanos B Molla, Allisyn C Moran, Kavita Singh, Florina Serbanescu, Andrew J Tatem, Kristine Nilsen

**Affiliations:** 1 Department of Social Statistics and Demography, University of Southampton, Southampton, UK; 2 MCSP, JHPIEGO, Washington, DC, USA; 3 GIS/IMMEL/ISD, American Red Cross, Washington, DC, USA; 4 MEL, Pathfinder International, Washington, DC, USA; 5 Department of Maternal, Newborn, Child and Adolescent Health, World Health Organization, Geneva, Switzerland; 6 MEASURE Evaluation/UNC, Chapel Hills, North Carolina, USA; 7 US Centers for Disease Control and Prevention, Atlanta, Georgia, USA; 8 Geography and Environment, University of Southampton, Southampton, UK; 9 WorldPop Research Group, University of Southampton, Southampton, UK

**Keywords:** geographic information systems, child health, maternal health, health services research, public health

## Introduction

Reproductive, maternal, newborn, child and adolescent health (RMNCAH) indicators, such as the maternal mortality ratio, often serve as a litmus test for health system performance, because women’s and children’s health lies at the core of any health system.^1^ The health and survival of women and children does not depend on a single intervention, but on packages of interventions delivered at all levels of the health system. Mapping and tracking RMNCAH therefore captures changes in wider health system performance. But mapping has traditionally been the domain of disease-specific tracking, providing useful, but limited snapshots of progress embedded in vertical intervention mechanisms. Although disease-specific spatial mapping and research are effective ways to identify geographic inequities and to inform service provision, geographic and spatial analyses of RMNCAH have the potential to provide a broader perspective. But such analyses, especially for routine RMNCAH care provision, have been underused despite their potential to inform programmes and policies in low/middle-income countries. This commentary also argues that visualisation of RMNCAH data provides a potent social accountability and decision-making tool. Given the topic’s importance, a supplement on the use of geographic information systems (GIS) in RMNCAH is long overdue.

Most geospatial studies in RMNCAH start with the geographical placement of facilities and facility staff—the fundamental infrastructure of all health systems—and the location of communities. Clearly, tackling frequently occurring birth and childhood health emergencies requires accessible, person-centred systems located near the client.^2^ Previous geospatial studies that have focused on determining access or distance to facilities have benefited from analytical techniques developed in the environmental science and physical geography fields. Few previous studies consider realistic travel over terrain or health facility ‘bypassing,’ where healthcare-seeking clients choose not to use their closest facility. This supplement thus breaks new ground by demonstrating that geospatial analysis has the potential to consider multiple important factors when assessing access to services including socioeconomic, financial, geographical and empowerment-related factors.^3^ Also important are measures of travel time to preferred facility and time to referral to higher level facilities.^4^

A recent literature review suggests there has been an increase in the number of studies exploring RMNCAH within an explicitly spatial context.^3^ GIS is also increasingly used to examine the relationship between maternal and child outcomes and the sociocultural environment,^5^ widening applicability in maternal and child health research. Spatial analysis can further uncover subnational-level differences in service coverage and health outcomes,^6^ which are often masked in aggregated national-level data sets. Mapping can be an important tool when used at the national and subnational levels for health service management and service planning.^7^

## Aiming for utopia

The papers in this supplement represent a much-needed step forward in GIS-based science for RMNCAH. However, to fully exploit the data and methods revolution that has been catalysed by the availability of new open-source tools for geospatial analysis, which are now accessible to low-resource settings, we need a vision for a GIS ‘utopia’ in RMNCAH. This commentary asks, ‘What would this utopia look like?’ As table 1 illustrates, we need progress on many fronts, from better data to better analytics along the impact chain from inputs to impact.

**Table 1:** Moving towards utopia: matrix of geographic information system developments needed for reproductive, maternal, newborn, child and adolescent health

	Data		Analytics			
Domain	Stronger protocols and guidance for data collection and data management	More accurate denominators	Thematic mapping	Spatial analysis	Spatial modelling	Live systems
Description	More temporally accurate and spatially referenced data on health facilities, health workers and health events	Better temporally and spatially referenced, high-resolution denominators	Creation of better maps to convey information about a topic or theme	More sophisticated analytics to extract or create new information from spatial data	Better spatial analysis that includes the use of mathematical models to simulate natural or anthropogenic phenomena	Live GIS systems used for day-to-day management of health service provision or in web-based apps
Developments and improvements needed along the impact chain						
Start of impact chain (inputs)	Censuses on place names, placement and characteristics of health facilities, for example, constantly updated open access master facility list; budget/financial health spending information and commodities tracking	Catchment area populations based on better quality and/or more frequent censuses and administrative data	Use of better data visualisations that have the appropriate content and level of detail for the target audience(s), including use of cartograms, and so on, for all stages in the impact chain	Use of continually updated GIS layers for settlements, rivers, physical landmarks, road/path networks and health facilities	Modelling to ‘fill in the gaps’ of health management information systems by extrapolation, to give approximate overview mapping of health system inputs	Continually updated information on facilities in web-based systems so that tracking can be live; commodity tracking can be linked to live systems as well as budget spending
Processes/quality of care outputs	More detailed, publicly available data on human resources, health management information system staff; RMNCAH laboratory screening tests and services received by clients; working with local communities to get place names	Population/demographic information, pregnancies, by age, gender (children), ethnicity, wealth status		Health management information system data to be used and analysed more frequently to produce new maps of health system processes	Human resource migration and forecasting for planning, based on location of doctors, midwives, nurses and training institutions	Quality indicators can be continually or at least regularly monitored as trends can develop quickly
Outcomes and impacts (morbidity and mortality)	Improvements in death registration and cause of death needed; improved ways to locate beneficiaries	Civil registration improvements are needed for accurate denominators		Death review mapping, and use of other outcome data to be mapped, for example, caesarean section, disease incidence	Production of outcome surfaces—mortality, complications and individual/complications/diseases/ fertility; better documentation of methods	Notifiable deaths and other key outcomes can be included in live systems

In an outline of the current state of the geography of maternal and newborn health, Ebener *et al*^3^ broadly categorised the published use of GIS methodology into three themes by increasing complexity: (1) thematic mapping (creation of basic maps to convey a topic or theme); (2) spatial analyses (creation or extraction of new information from spatial data); and (3) spatial modelling (spatial analysis with the use of mathematical or statistical models to simulate real-world phenomena).

GIS, geographic information system; RMNCAH, reproductive, maternal, newborn, child and adolescent health.

Improvements are needed across the inputs, processes/quality of care outputs and outcomes/impact spectrum, and across UN Global Strategy domains—for example, GIS for humanitarian disasters, determinants of health for intersectoral use and analyses related to adolescent health.^8^ However, a GIS utopia demands more than progress. By the end of the Sustainable Development Goals (SDG) era, we envision ministries of health, non-governmental organisations and universities in all countries regularly using GIS applications to create maps that display routine and periodic health data at national and subnational levels that will aid in evidence-based planning strategies. We aim for nothing less than the routine use of such maps to inform policy development and programme management decisions, ultimately helping to reduce inequities in health service access, intervention coverage and health outcomes. These maps and other health information, such as data captured in national and district RMNCAH scorecards, will be made publicly available to foster transparency and accountability among stakeholders for progress towards national and global health goals.

Illuminating inequity is a key part of our vision for GIS in RMNCAH, but more is possible. Presenting information in a visually engaging and easy-to-understand format can facilitate data-driven decisions. As the world becomes increasingly paperless and the hunger for real-time data for strategy and accountability escalates, the use of data visualisation to organise and share information has grown. GIS analyses and tools help to present data visually so that they can be more readily comprehended by diverse audiences. However, capacity to understand and act on the data may still need to be strengthened in some settings.

## Seizing opportunities

Due in part to worldwide initiatives, such as the SDGs, which encourage evidence-based development, quality spatial and geographic data are increasingly available in low/middle-income countries. This development presents opportunities to take a geographic perspective for planning and strategic decision-making in global health initiatives and in RMNCAH. These opportunities involve riding the wave of emerging data availability and data analytics tools as well as engaging with large and small-scale initiatives, such as the Health Data Collaborative, which is an effort launched in 2015 by multiple global health partners to work with countries to improve the availability, quality and use of data for local decision-making and tracking progress towards the health-related SDGs.

Periodic data sources remain key to progress. As data collection moves from paper forms to mobile digital tools that can capture GIS coordinates, and health facility assessments and household surveys routinely incorporate geolocators, new analyses will be possible, including linking household and survey data. Mapping of health service statistics (national health management information system and logistics management information system data) and health facility assessment data by district presents new opportunities to examine spatial patterns in the quality of RMNCAH care. This mapping could potentially assist ministries of health to better target scarce resources for supportive supervision/mentoring and in-service training of health workers.

Data collection and analytics are also seeing more sophisticated usage as more open-source, user-friendly tools, such as Open Data Kit (https://opendatakit.org/) and QGIS (https://qgis.org), become available. Concurrently, ministries of health in low/middle-income countries are adopting electronic information systems, which has helped to increase the availability of routine health management information system data (District Health Information System 2, among the most widely used of these systems, even includes basic mapping functions).

One of the most important public health lessons from recent years is that timely information, including spatial information, is key to averting emerging disease threats, such as Zika and Ebola, and addressing humanitarian situations. Having health-related geographic information is central to creating resilient health systems. Wide acknowledgment of this reality can help us garner resources to scale up GIS capability.

## Addressing the gaps

This supplement is part of the movement to seize opportunities to move towards a GIS utopia in RMNCAH. Other initiatives, such as capacity strengthening, websites that facilitate data sharing and communities of practice, are also addressing gaps in use of GIS for RMNCAH data. Table 2 lists some examples.

**Table 2:** Existing global strategies to address gaps in geographic information systems for reproductive, maternal, newborn, child and adolescent health

Training initiatives	Communities of practice (COP)	Creating an evidence-based culture	Data exchange
The Demographic and Health Surveys programme trains Ministry of Health (MOH) partners on GIS when implementing surveys. MEASURE Evaluation does GIS training (45 countries to date): https://www.measureevaluation.org/resources/training/capacity-building-resources/geographic-information-systems-mapping-and-analysis-of-spatial-data/gis-training eLearning courses available at: https://www.globalhealthlearning.org/course/gis2https://www.globalhealthlearning.org/course/geographic-approaches-global-health	COPs for GIS education and ongoing professional support: https://www.tandfonline.com/doi/pdf/10.1080/03098265.2017.1315534?needAccess=true MEASURE Evaluation GIS Working Group: meets annually: https://www.measureevaluation.org/resources/publications/ws-17-41 Open Data Kit community forum to better understand how to contribute/improve spatial data collection for your project and programmes: https://forum.opendatakit.org/ HOT is an international team dedicated to providing open community map data which aids in emergency response, reducing risks and contributing to SDG goals: https://www.hotosm.org/what-we-do	Use of Spatial Quality and Anomalies Diagnosis (SQUAD) tool to analyse spatial data for accuracy and to chart a path for improving data: https://www.measureevaluation.org/resources/publications/ms-18-146 Norwegian Agency for Development Cooperation ‘Mapping for MNH’	Humanitarian data exchange is an open platform for sharing data across crises and organisations: https://data.humdata.org/ WHO Global Health Observatory data repository contains data that can be linked to GIS shapefiles: http://apps.who.int/gho/data/node.home Gridded Population of the World (GPW), NASA, models the distribution of human population on a continuous global raster surface: http://sedac.ciesin.columbia.edu/data/collection/gpw-v4 WorldPop Geospatial Data Sets: high spatial resolution data on human population from the WorldPop group at University of Southampton: www.worldpop.org Demographic Health Survey (DHS) Program’s Spatial Data Repository provides geographically linked health and demographic data from the DHS Program and the US Census Bureau for mapping using GIS: https://spatialdata.dhsprogram.com/home/ OpenStreetMap is built by a community of mappers that contribute and maintain data about roads, trails, cafés, railway stations and much more, all over the world: https://www.openstreetmap.org/about Global Open Source Repository for Health Facilities: Global Health Sites Mapping Project is building a curated open data commons of health facility data with OpenStreetMap: https://open-proposals.ucsf.edu/digital-square/notice-b/proposal/14307 Healthsites.io is also building an open data commons of health facility data with OpenStreetMap: https://healthsites.io/ Other developments: Increased availability of master facility lists. Standardising technology for interoperability.

GIS, geographic information system; HOT, Humanitarian OpenStreetMap Team; MNH, maternal and newborn health; NASA, National Aeronautics and Space Administration; SDG, Sustainable Development Goals.

## Conclusions

GIS data can reveal patterns and trends to support targeted interventions and resource allocation. However, at the moment, GIS-informed policies, planning and priority setting are rare, particularly in low-resource settings where many poor health outcomes occur. There is a need to expand GIS use and galvanise efforts to improve the spatial data platforms that could strengthen application of GIS methods. A clear recognition of the role of GIS in monitoring progress towards global targets provides greater understanding of how GIS data can inform decision-making. Furthermore, this recognition leads to continued improvements in the availability and quality of geocoded data, and improved local capacity to perform and interpret GIS analyses could accelerate use of these systems.
